# Multidisciplinary team efforts to improve the pregnancy outcome of pregnancy complicated with primary hyperparathyroidism: case series from a single hospital

**DOI:** 10.1186/s12884-021-04042-7

**Published:** 2021-08-22

**Authors:** Hai-ning Jiao, Li-hao Sun, Yan Liu, Jian-qiao Zhou, Xi Chen, Jian-min Liu, Hui-ping Zhong

**Affiliations:** 1 Department of Obstetrics and Gynecology, Ruijin Hospital, Shanghai Jiao Tong University School of Medicine, 197 Ruijin Er Road, Shanghai, 200025 China; 2 Department of Endocrine and Metabolic Disease, Ruijin Hospital, Shanghai Jiao Tong University School of Medicine, Shanghai, China; 3 Department of Ultrasonography, Ruijin Hospital, Shanghai Jiao Tong University School of Medicine, Shanghai, China; 4 Department of Surgery, Ruijin Hospital, Shanghai Jiao Tong University School of Medicine, Shanghai, China

**Keywords:** Multidisciplinary team, Pregnancy, Primary hyperparathyroidism, Prognosis

## Abstract

**Background:**

There is no consensus or management algorithm for primary hyperparathyroidism (PHPT) in pregnancy.

**Methods:**

This study comprises a retrospective case series. From August 2014 to December 2020, 9 cases of PHPT in pregnancy were diagnosed by a multidisciplinary team (MDT) consultation center of obstetrics in our hospital. Their clinical manifestations, treatment strategies, and maternal and infant outcomes were analyzed.

**Results:**

The median onset age of the patients was 32 (25 ~ 38) years. PHPT was diagnosed in two cases before pregnancy, in six cases during pregnancy and in one case postpartum. The main clinical manifestations were nausea, vomiting, and other nonspecific symptoms, with anemia as the most common maternal complication. Hypercalcemia crisis was developed in one case. The median levels of preoperative serum calcium and parathyroid hormone (PTH) were 3.08 (2.77 ~ 4.21) mmol/L and 300.40 (108.80 ~ 2603.60) pg/ml, respectively. The parathyroid ultrasonography tests were positive in eight cases and negative in one patient who had an ectopic lesion localized by ^99m^Tc-MIBI. Parathyroidectomy was conducted in 7 cases during the 2nd trimester, including 2 patients diagnosed before pregnancy who refused surgery, 1 patient during the 1st trimester, and 1 patient postpartum, with a significant reduction in serum concentrations of calcium and PTH. A management algorithm was developed.

**Conclusion:**

This case series suggests that pregnant women with PHPT should be managed by MDT according to the algorithm. If PHPT is confirmed in fertile women before pregnancy, parathyroidectomy should be strongly suggested and performed. If PHPT is diagnosed during pregnancy, even in its mild form, surgical treatment, optimally during the 2nd trimester, is effective and safe for pregnancy and neonatal outcome.

## Background

Primary hyperparathyroidism (PHPT) is an endocrine disorder characterized by high serum calcium levels, high parathyroid hormone (PTH) levels and low serum phosphorus concentration [1]. PHPT is the most common cause of hypercalcemia during pregnancy. Exposure to higher than normal levels of PTH can cause various hypercalcemia-related clinical manifestations, with an incidence of maternal complications of approximately 67% and that of fetal or neonatal complications of approximately 80%, leading to 30% fetal or neonatal deaths [2]. The clinical manifestations of PHPT during pregnancy are atypical, including nausea, vomiting, constipation, and fatigue, which overlap pregnancy reactions and are overlooked. Currently, the reports of PHPT in pregnancy are usually case reports or small series, and no consensus has been reached on the management of this special disorder [3].

The multidisciplinary team (MDT) is an effective medical model that integrates the advantages of multiple clinical specialists for the comprehensive diagnosis and treatment of diseases [4]. For the management of high-risk pregnancy patients with PHPT, physicians and surgeons from obstetrics, endocrinology, surgery and other disciplines should jointly evaluate the patient’s condition and determine the treatment plan. In the past 20 years, our hospital, as a tertiary university teaching hospital, has accumulated some experience in the diagnosis and treatment of PHPT [5–7]. To improve the care of PHPT in pregnancy, this study summarizes the clinical features, diagnosis and treatment experience of this disorder at our MDT center and proposes a diagnosis and treatment algorithm for this disease.

## Patients and methods

### Patients

We retrospectively reviewed 11 pregnant patients with PHPT between August 2014 and December 2020 at our MDT consultation center of obstetrics. This study was approved by the Ethics Committee of Ruijin Hospital at Shanghai Jiaotong University (KY2020–100). Among these 11 pregnant patients with PHPT, 10 patients conceived naturally, and 1 patient used in vitro fertilization (IVF). To analyze the effect of PHPT during pregnancy, including the effect of surgery during pregnancy on disease prognosis, we excluded two patients who underwent parathyroid surgery after induced labor.

The median onset age was 32 (25 ~ 38) years. There were six cases of primipara and three cases of pluripara (including one case of a scarred uterus). Two patients came to our center for consultation due to a history of missed abortion and were diagnosed with PHPT. Surgical treatment was recommended, but both patients were reluctant to undergo surgery and received follow-up care. However, both patients had to undergo surgery during their 2nd trimester. One case of PHPT was retrospectively diagnosed by us 5 months postpartum because the baby was born with hypocalcemic convulsions at another hospital. Her serum calcium level was increased during pregnancy, but no particular management was taken. The other six patients were diagnosed with PHPT in their second trimester. The study protocol was approved by the Institutional Review Board of the Rui-jin Hospital in Shanghai.

### Methods

MDT: Two rounds of multidisciplinary (obstetrics, endocrinology, surgery, ultrasound, anesthesiology, intensive medicine and others) discussions were conducted. The first discussion was held when the patients visited for the first time (usually during the first trimester or second trimester) to confirm the diagnosis and determine whether they should be treated surgically. The second discussion was initiated during the patient’s late pregnancy (38 weeks of pregnancy) to determine the timing and method of delivery. We encouraged patients to have a vaginal delivery. For patients with obstetrical factors, a cesarean section at 39 weeks of gestation pregnancy was recommended. All the decisions were fully communicated with the patients and their family members.

Laboratory tests: For all pregnant women with suspected PHPT, routine blood tests, hepatic and renal function, serum electrolytes, PTH (reference range: 15–68.3 pg/ml; 8 K25 ARCHITECT intact PTH; Abbott Diagnostics) and 25OHD (reference range: 50 nmol/L; Elecsys and Cobas E analyzer; Roche Diagnostics) were measured. If the patient’s serum calcium or albumin adjusted calcium level increased with the elevation of serum PTH after excluding secondary hyperparathyroidism due to renal failure or vitamin D deficiency, the diagnosis of PHPT in pregnancy was made.

Serum osteocalcin (OC), β-carboxy-terminal telopeptide of type I collagen (CTX) and procollagen type I N-terminal propeptide (PINP) were measured (OC reference range: 1.8 ~ 8.4 ng/ml, PINP reference range: 15.13 ~ 58.59 ng/ml; CTX reference range: 0.025 ~ 0.573 ng/ml, Cobas 601, Roche Diagnostics).

Imaging tests: All pregnant women underwent neck ultrasound. 99mTc-MIBI single-photon emission computed tomography (SPECT/CT) was only performed in patients before pregnancy (*n* = 1), in patients with no intention to continue pregnancy (n = 1, 2nd trimester) and in patients after labor (n = 1).

Follow-up: Follow-up was conducted for postoperative pregnant patients with PHPT, either by face-to-face consultation or phone interview, to monitor conditions including postoperative blood electrolytes, neonatal blood electrolyte levels and complications.

Statistical analysis: Normally distributed data are expressed as the means ± SD, while nonnormally distributed data are expressed as medians (range). All the data were processed using SAS 9.0 statistical software (SAS Institute Inc). *P* < 0.05 was considered to be statistically significant.

## Results

### Clinical manifestations and maternal complications

The main symptoms were nausea, vomiting, fatigue, anorexia, and other pregnancy reactions, which were present in 6 of 9 patients (66.67%) in early pregnancy. Anemia was present in four patients (44.44%), with Hb levels ranging from 84 g/L to 103 g/L. One patient (11.11%) developed a hypercalcemia crisis.

Two patients had bilateral knuckle and knee joint pain in early pregnancy; bone pain was also noticed in another patient (case 1) who did not undergo surgery after delivery, with multiple osteoporotic lesions in the ilium, femur and spine as revealed by MRI.

The general characteristics and clinical symptoms of the patients are outlined in Table 1.

**Table 1:** Clinical manifestation and maternal complications of pregnant patients with PHPT

Case	Time of diagnosis	Clinical manifestation	Maternal complication
1	5 months postpartum	No obvious manifestations	Multiple bone destruction and osteoporosis
2	before pregnancy	Bilateral knuckle and knee joint pain	Anemia (99 g/L)
3	2nd trimester	nausea, vomiting, fatigue, anorexia	Anemia (89 g/L)
4	2nd trimester	nausea, vomiting,	Anemia (84 g/L), hypercalcemia crisis with renal failure
5	before pregnancy	nausea, vomiting, fatigue, anorexia	None
6	2nd trimester	nausea, vomiting, fatigue, anorexia	Asymptomatic left kidney stone
7	2nd trimester	nausea, vomiting, fatigue, anorexia	Anemia (103 g/L)
8	2nd trimester	Bilateral knuckle and knee joint pain	None
9	1nd trimester	nausea, vomiting, fatigue, anorexia	None

### Biochemical and imaging tests

The median serum calcium level was 3.08 (2.77 ~ 4.21) mmol/L, with a median serum PTH level of 300.40 (108.80 ~ 2603.60) pg/ml. The median serum 25OHD concentration was 22.27 (13.50 ~ 39.45) nmol/l. For the bone biochemical markers, the serum OC level was 7.60 (3.92 ~ 14.10) ng/ml, the PINP level was 85.43 (65.69 ~ 233.7) ng/ml, and the serum CTX level was 0.53 (0.4 ~ 1.73) ng/ml (Tables 2 and 3).

**Table 2:** Preoperation biochemical markers of pregnant patients with PHPT

Case	Ca (mmol/L)	P (mmol/L)	ALP (IU/ml)	Cr (μmol/L)	Albumin (g/L)	RBC (× 10^**9**^/L)	HGB (g/L)
1	2.87	0.64	538	37	39	4.63	144
2	2.95	1.04	59	54	28	2.94	99
3	3.28	0.65	59	56	36	2.89	89
4	4.21	1.39	84	223	31	2.74	84
5	2.77	0.69	54	43	37	3.6	117
6	3.00	0.66	176	47	36	4.32	139
7	3.49	0.59	78	29	33	3.86	103
8	3.17	0.81	85	39	37	3.83	126
9	3.08	0.72	120	42	45	3.76	115

**Table 3:** Preoperation bone biochemical markers of pregnant patients with PHPT

Case	PTH (pg/ml)	25-OH-VitD (nmol/L)	OC (ng/ml)	PINP (ng/ml)	CTX (ng/ml)
1	1524.1	13.50	12.00	233.70	1.73
2	108.8	20.69	6.50	212.60	0.90
3	300.4	39.45	7.60	83.52	0.53
4	2603.6	38.63	7.53	79.20	0.57
5	201.0	25.12	9.20	85.43	0.52
6	494.3	22.27	3.92	65.69	0.40
7	208.9	16.68	4.68	127.20	0.44
8	158.2	35.35	14.10	85.52	0.97
9	305.2	19.82	8.19	73.41	0.29

Neck ultrasonography revealed the presence of low-density lesions in 8 of 9 cases. 99mTc-MIBI SPECT/CT imaging was conducted in three patients. Low-density foci suggestive of a parathyroid lesion were revealed by MIBI in all cases, including one ectopic lesion anterior superior mediastinal (posterior right of the sternum hilum) in one patient.

All 9 patients underwent kidney, ureteral, ureter, and adrenal ultrasonography, and only 1 patient was found to have a left kidney stone with a diameter of approximately 8 mm.

### Treatment of pregnant patients with PHPT

Preoperative management with oral or intravenous administration of large amounts of normal saline was adopted to reduce the serum calcium concentration. Calcitonin and furosemide were not administered to pregnant patients.

**Fig. 1:**
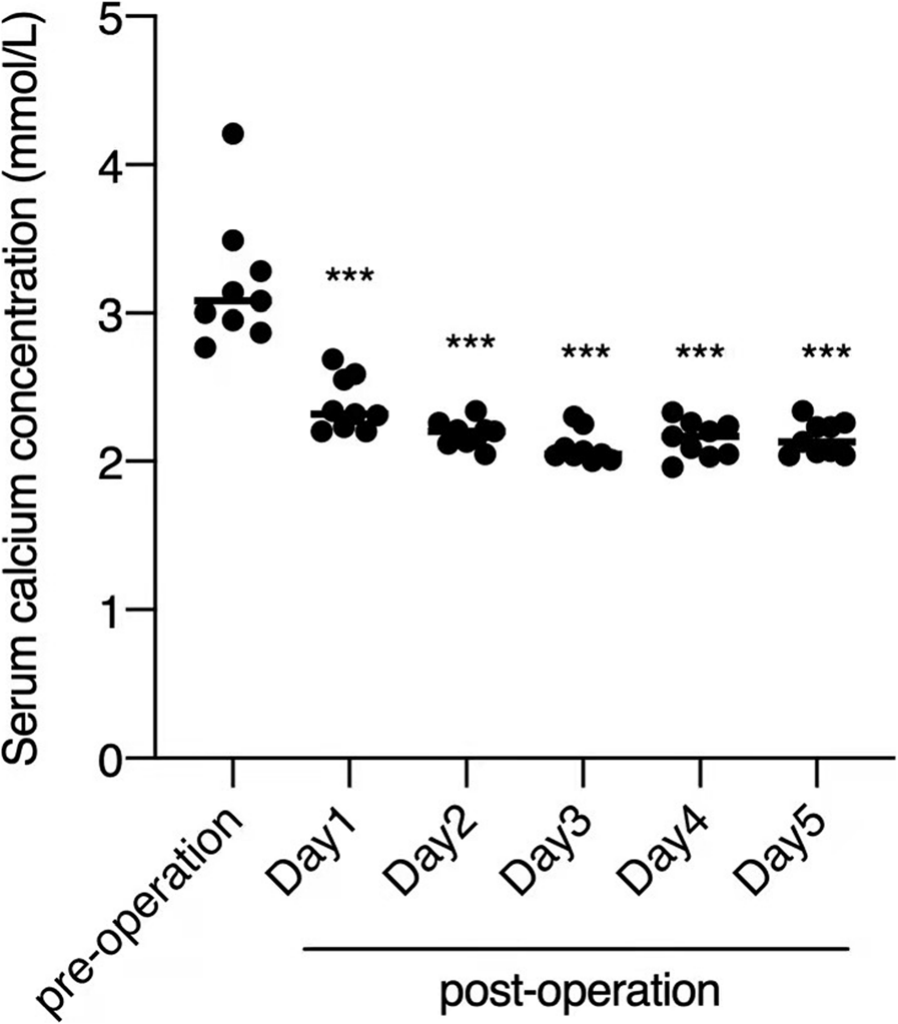
Preoperation and postoperation serum calcium levels (mmol/L) in pregnant patients with PHPT

Parathyroidectomy was performed uneventfully in all 9 cases with general anesthesia, including 7 patients who underwent surgery during the 2nd trimester (9 ~ 26 weeks), 1 patient who underwent surgery during the 1st trimester and 1 patient who underwent surgery postpartum. The median operation time was 65 mins (50 ~ 80 mins), and the median blood loss was 50 ml (20 ~ 1100 ml). Parathyroid surgery was successful in all cases, and no complications occurred. The median percentage decrease of PTH on the first day after parathyroid surgery was 95.49% (72.95% ~ 99.65%). The serum calcium concentrations in these patients gradually decreased to the lowest levels on the 3rd and 4th days after the operation (Fig. 1). Calcium/vitamin D (Caltrate) supplementation and/or an active vitamin D analog (Calcitriol) was prescribed according to the serum calcium levels.

Postoperative pathology examination showed single parathyroid adenoma in eight cases and parathyroid carcinoma in one case.

### Pregnancy outcome and neonatal complications

There was one case of patient-requested labor induction in this series. The remaining eight cases were full-term pregnancies. Among them, three cases were vaginal deliveries, and five cases were terminated by cesarean section due to obstetric indications (scarred uterus, macrosomia, cephalopelvic disproportion, and twin pregnancy).

For patients undergoing parathyroidectomy during pregnancy at our hospital, the Apgar scores for all the infants at 1 and 5 min after birth were 10 points, with no distinct abnormality. However, one child was diagnosed with autism at the age of 4 (Table 4).

**Table 4:** Pregnancy outcome and neonatal complications of pregnant patients with PHPT

Case	gestational age at delivery(weeks)	delivery mode	birth weight(g)	neonatal complications
1	39^+ 1^	vaginal delivery	2800	The baby was born with hypocalcemic convulsions
2	39	cesarean section	3455	The child was diagnosed with autism at the age of 4
3	39^+ 2^	cesarean section	3075	normal
4	14	labor induction	/	/
5	38^+ 2^	vaginal delivery	2910	normal
6	39^+ 5^	vaginal delivery	3310	normal
7	39	cesarean section	3620	normal
8	39^+ 2^	cesarean section	3690	normal
9	39^+ 1^	cesarean section	3110	normal

## Discussion

### Clinical manifestations and diagnosis of pregnant patients with PHPT

PHPT is characterized by elevated serum PTH concentration and hypercalcemia [8], with varying degrees of severity from asymptomatic to hypercalcemic crisis [9, 10]. Among pregnant patients with PHPT [11], 80% develop nausea, vomiting, and frequent (nocturnal) urination, which overlap with normal physiological reactions during pregnancy and are easily overlooked, complicating or delaying the diagnosis of PHPT [11]. Similar findings were also noticed in our series. The high percentage of anemia detected in our series was due to either PHPT [5] or physiological hemodilution during pregnancy.

The use of the albumin-adjusted serum calcium level [11, 12] is important in patients with low serum albumin levels, especially in pregnant women who may have a 10% decrease in serum calcium level [11]. However, in our series, all women with PHPT still had an elevated serum calcium level (median: 3.08 mmol/L). In China, although the clinical and biochemical patterns of PHPT are becoming milder, PHPT is still more severe than in Western countries [5, 6], which seems to also be the case for pregnant women with PHPT.

Postoperative pathology showed one case (case 4) of parathyroid carcinoma with a serum calcium level of 4.21 mmol/L and serum PTH level of 2603.6 pg/ml. Parathyroid carcinoma is a rare cause of PHPT, accounting for less than 1% of Western PHPT patients and 6% of Chinese patients [5]. However, in this series, parathyroid carcinoma accounted for 11.1% (1/9), and we are not sure whether such a high rate in pregnant women has any physiological and pathophysiological relationship with pregnancy. The clinical manifestations of parathyroid carcinoma are mainly moderate to severe hypercalcemia and symptoms of renal and skeletal involvement. Whether malignant tumors are considered at the time of initial diagnosis is critical for the patient’s prognosis. Serum calcium and serum PTH levels in patients with parathyroid carcinoma are significantly higher than those in parathyroid adenoma. When serum calcium exceeds 12 mg/dL (3 mmol/L), serum PTH levels exceed the normal upper limit by 3–10 times, and awareness of the possibility of parathyroid carcinoma is necessary [13]. The clinical symptoms of parathyroid carcinoma are usually more severe than those of parathyroid adenoma and often involve the kidneys and bones. If the patient has severe renal and skeletal complications, the possibility of malignancy should be considered.

The method for determining the location of parathyroid lesions in pregnancy with PHPT differs from that of ordinary PHPT; the commonly applied 99mTc-MIBI and neck CT examinations are limited during pregnancy. The sensitivities of 99mTc-MIBI and color Doppler neck ultrasonography to localize parathyroid lesion(s) are 94.1 and 85.1%, respectively; if they are combined, the sensitivity will increase to 98.9% [14].

The use of 99mTc-MIBI in pregnant women remains controversial [15]. A case report [16] suggested that pregnancy is not a contraindication to 99mTc-MIBI examination due to the short half-life of the radionuclide, ensuring the safety of 99mTc-MIBI examination, especially when ultrasound cannot accurately localize the lesion during pregnancy [17]. However, in another case series, the authors suggested avoiding the use of radioactive imaging modalities [15]. In our series, 99mTc-MIBI was only employed in patients before conception or with no intention to continue pregnancy or in patients after labor, with 100% positive findings. An ectopic lesion was fortunately and coincidently revealed by MIBI in one case (case 1), which was not identified by ultrasound. However, due to the lack of solid clinical data, the use of 99mTc-MIBI in pregnant women with the intention to continue pregnancy should be cautious.

According to our results, neck ultrasonography can localize all the parathyroid lesions in the neck in 8 cases, except an ectopic lesion. The accuracy of the ultrasound positioning was further confirmed during surgery. From our experience and other similar case reports [18], neck ultrasound for pregnant patients with PHPT is recommended as the first choice of treatment and is effective for preoperative positioning and surgical guidance.

### Treatment of pregnant patients with PHPT

For pregnant patients with PHPT with asymptomatic, mildly elevated, serum calcium levels or patients who do not accept surgery, close observation without surgical intervention is feasible [19, 20]. The first-line medication is oral or intravenous rehydration with or without furosemide, which is safe, can avoid iatrogenic placental hypoperfusion or oligohydramnios caused by dehydration [21] and will not increase the risk of obstetric complications, such as abortion. However, there is still a need to closely monitor the serum calcium level and disease progression.

PHPT is usually caused by a single parathyroid tumor, and the most effective treatment is surgery [22]. For pregnant women, surgery should be implemented in the second trimester (13 ~ 26 weeks of gestation) because the fetal organs have completely developed and the spontaneous abortion rate is the lowest at this point [23], effectively reducing the risk of maternal and fetal complications. A study of 77 pregnant women revealed that even if serum calcium is maintained at a median level of approximately 2.67 mmol/L, a 12% risk of fetal death still exists [24]; if the serum calcium level is sustained at 2.70 ~ 2.75 mmol/L, the risk of fetal death will increase [25]. It is suggested that PHPT patients with a history of miscarriage and a serum calcium level exceeding 2.75 mmol/L, even without distinct elevation or symptoms, should undergo surgery and should not wait until postpartum to avoid neonatal hypocalcemia and convulsion [26].

From our experience and that of others [15], parathyroidectomy conducted in the 2nd trimester, especially when guided by experienced MDT, is safe and effective. However, for patients with serious hypercalcemia, such as hypercalcemia crisis (case 4) or an uncontrollable elevated serum calcium level (case 9), timely surgery is still needed [27], even in the 1st trimester (case 9) in our series.

In nonpregnant PHPT patients, the parathyroid lesion could be confirmed to be removed if the serum PTH level was decreased more than 50 and 60% at 5 min and 15 min, respectively, after lesion resection. Intraoperative PTH was measured in our series in two cases (case 8 and case 9), showing a 65.53% ~ 78.19% decrease 10 min after parathyroidectomy.

Surgical therapy is also the most frequent option for the treatment of parathyroid carcinoma. Primary operation is crucial for adequate local excision. The tumor was removed in one block with ipsilateral thyroid gland lobectomy. During the operation, the surgeon should avoid rupture of the capsule. It would be preferable to perform an ipsilateral thyroid gland lobectomy with parathyroid tissue and neck block dissection [28].

It should be stressed that PHPT can be diagnosed biochemically, not necessarily to be confirmed by localization imaging results [29]. For cases with inconsistent or negative imaging findings, if surgery is indicated, the patient should still undergo surgery. For patients with positive preoperative localization, focused parathyroidectomy could be performed, while for patients with negative imaging results, bilateral cervical exploration (BCE) could be performed, which has a cure rate in excess of 95% for experienced surgeons [30, 31].

Notably, there was a case of PHPT in our patient (case 2) diagnosed 4 years before pregnancy at our hospital. Surgery was recommended, although her serum calcium level was at the normal upper limit. However, the patient refused the operation. During pregnancy, her serum calcium level increased to 2.95 mmol/L, and she had to undergo surgery at 23^+ 5^ weeks of gestation, with a normal delivery at full term. However, her child was diagnosed with autism at the age of 4. Because the child’s father had premature Parkinson’s disease, we are not sure whether a relationship exists between this child’s autism and his parents’ diseases. However, based on our experience with this case, we believe that whenever a diagnosis of PHPT is made for women of reproductive age, surgery should be strongly suggested to the patients.

Other methods are available to manage parathyroid lesions in pregnant women. Alcohol ablation could be considered in the case of ineffective drug therapy or when a pregnant woman cannot tolerate surgery [32, 33]. This method was not attempted in our series.

### Timing and method of pregnancy termination of patients with PHPT during pregnancy

Presently, no consensus exists on the timing and delivery method of pregnancy termination in patients with PHPT. Vaginal delivery can be performed under close supervision, but obstetric factors should be considered first. After the diagnosis of pregnancy with PHPT, we fully communicated with the patients and their families during the 2nd trimester and reached a consensus on pregnancy intention (labor induction or continued pregnancy), parathyroid surgery treatment and surgery timing. One patient was fertile and had concerns about the safety of the fetus after parathyroid surgery and requested the termination of pregnancy. There were eight cases of full-term pregnancy, among which three cases were delivered naturally under close monitoring and four cases were terminated by cesarean section under lumbar anesthesia at approximately 39 weeks of gestation due to obstetric factors.

### Screening and perinatal production inspection patterns for pregnant patients with PHPT

Pregnancy with PHPT can cause severe maternal and infant complications, especially the development of hypercalcemia crisis, which can lead to perinatal maternal death. With our previous experience in PHPT diagnosis and treatment [5–7], we suggest that serum electrolytes should be checked during prepregnancy examination for fertile women. If the serum calcium level is higher than 2.43 mmol/L [5], serum PTH testing and other tests should be performed for PHPT screening. If any abnormality occurs, an endocrinologist should be consulted as soon as possible.

**Fig. 2:**
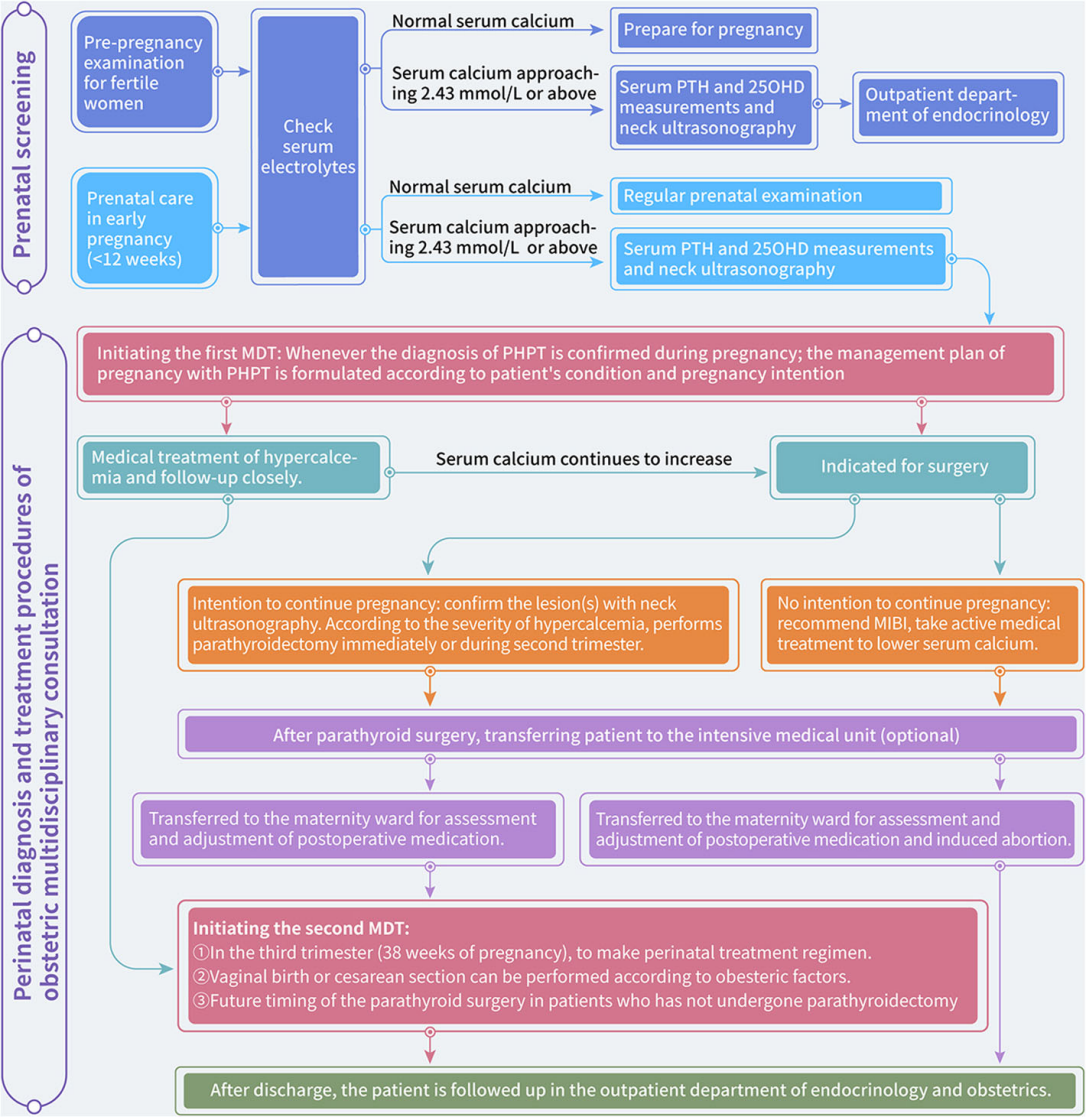
Prenatal screening and perinatal diagnosis and treatment procedures of pregnancy with PHPT at Ruijin Hospital

We have developed an obstetrical screening program for pregnant women. In early pregnancy (< 12 weeks), the serum electrolyte levels are checked at the hospital during the initial diagnosis. If abnormalities in serum calcium occur, serum PTH testing and other tests are performed for PHPT screening. For suspected cases, MDT consultation is made in time to develop a treatment plan. For patients with PHPT during pregnancy, postoperative monitoring of parathyroid function should be strengthened. In addition to routine parameters, serum calcium and PTH concentrations should be checked every 2 weeks, and postoperative medication should be adjusted to avoid hypocalcemia. The obstetric MDT process for patients with PHPT is illustrated in Fig. 2.

### Limitations

There are several limitations in this study. Due to the rareness of PHPT in pregnancy, the sample size in this study was small. Some follow-up data regarding patients’ and their children’s serum calcium levels were obtained through phone interviews. Intraoperative PTH was not routinely measured in any of our patients. PTH monitoring during surgery is helpful to determine the success of the operation, especially for cases with negative preoperative localization [34] or multiple adenomas [35]. We did not routinely test for the MEN-1 gene, as in other reports [12, 34]. The test was performed in only one case with a family history in our series, and the result was negative.

## Conclusions

Clinical manifestations of PHPT in pregnancy are atypical and prone to be overlooked. It is recommended to focus on serum electrolyte levels during the first trimester, especially in pregnant women with significant nausea and vomiting. If the serum calcium level is increased, the serum PTH level should be checked to confirm the diagnosis of PHPT, and then an MDT should be organized to make follow-up and treatment plans. If PHPT is diagnosed before pregnancy in women of fertile age, even if it is a mild or asymptomatic case and the patient is reluctant, parathyroidectomy should be strongly recommended. If PHPT is diagnosed during pregnancy, considering the general accuracy of neck ultrasonography examination, timely parathyroidectomy, optimally in the second trimester of pregnancy or even in the first trimester, is effective and safe.

## Data Availability

The research data used to support the findings of this study were supplied by Prof.Zhong under license and so cannot be made freely available. Requests for access to these data should be made to Prof.Zhong (Email:zhp10392@rjh.com.cn).

## Abbreviations

PHPT: primary hyperparathyroidism
PTH: parathyroid hormone
MDT: multidisciplinary team
IVF: in vitro fertilization
OC: serum osteocalcin
CTX: β-carboxy-terminal telopeptide of type I collagen
PINP: procollagen type I N-terminal propeptide
SPECT/CT: 99mTc-MIBI single-photon emission computed tomography
MRI: magnetic resonance imaging
